# Mechanisms Driving Palmitate-Mediated Neuronal Dysregulation in the Hypothalamus

**DOI:** 10.3390/cells10113120

**Published:** 2021-11-11

**Authors:** Calvin V. Lieu, Neruja Loganathan, Denise D. Belsham

**Affiliations:** 1 Department of Physiology, University of Toronto, Medical Sciences Building 3247A, 1 King’s College Circle, Toronto, ON M5S 1A8, Canada; Calvin.Lieu@mail.utoronto.ca (C.V.L.); neruja.loganathan@mail.utoronto.ca (N.L.); 2 Departments of Obstetrics/Gynecology and Medicine, University of Toronto, Toronto, ON M5S 1A8, Canada

**Keywords:** palmitate, hypothalamus, energy homeostasis, reproduction, circadian rhythm, microRNAs, leptin, insulin, NPY, POMC

## Abstract

The hypothalamus maintains whole-body homeostasis by integrating information from circulating hormones, nutrients and signaling molecules. Distinct neuronal subpopulations that express and secrete unique neuropeptides execute the individual functions of the hypothalamus, including, but not limited to, the regulation of energy homeostasis, reproduction and circadian rhythms. Alterations at the hypothalamic level can lead to a myriad of diseases, such as type 2 diabetes mellitus, obesity, and infertility. The excessive consumption of saturated fatty acids can induce neuroinflammation, endoplasmic reticulum stress, and resistance to peripheral signals, ultimately leading to hyperphagia, obesity, impaired reproductive function and disturbed circadian rhythms. This review focuses on the how the changes in the underlying molecular mechanisms caused by palmitate exposure, the most commonly consumed saturated fatty acid, and the potential involvement of microRNAs, a class of non-coding RNA molecules that regulate gene expression post-transcriptionally, can result in detrimental alterations in protein expression and content. Studying the involvement of microRNAs in hypothalamic function holds immense potential, as these molecular markers are quickly proving to be valuable tools in the diagnosis and treatment of metabolic disease.

## 1. Introduction

### 1.1. Obesity and Palmitate Consumption

The World Health Organization has estimated that nearly 2 billion people worldwide are obese or overweight, which represents a near tripling of the obesity rate since 1975 [1]. Obesity is accompanied by dangerous comorbidities such as type-2 diabetes mellitus, cardiovascular disease, infertility, and some cancers [2,3]. The primary cause of obesity is positive energy balance wherein individuals consume more energy than is expended. Another important factor in the development or predisposition to obesity is the source of nutrients contributing to the positive energy balance. The increase in obesity rates has coincided with a 50% increase in the consumption of fats [4], and diets high in fat (HFD) induce metabolic syndrome in rodents and mice [5]. There are two general categories of fatty acids: saturated fats, which are commonly called “bad” fats, and unsaturated fats, which are considered “healthy” fats. The increase in dietary fat consumption has been primarily in the form of saturated fatty acids, which make up half of the fatty acid content in palm oil, the most commonly consumed cooking oil in the world.

Palmitate is a 16-carbon saturated fatty acid that is crucial for cellular function, since it is a component in the synthesis of membrane phospholipids, transport lipids, and palmitoylated proteins [6]. Although palmitate is important for cellular function and is synthesized endogenously via de novo lipogenesis, the excessive consumption of palmitate can have adverse consequences [7]. Under normal circumstances, palmitate is converted to triglycerides for long-term energy storage, but excess palmitate leads to increased production of potentially detrimental palmitate metabolites [8,9]. Palmitate and its metabolites can induce several forms of cellular stress, including endoplasmic reticulum (ER) stress, oxidative stress, and inflammation [10,11]. This is particularly a problem for the hypothalamus as obese individuals have higher concentrations of palmitate in their cerebrospinal fluid (CSF) [12] and brain [13]. This review will focus on the disruptive effects of palmitate on the hypothalamic control of basic physiological processes, the mechanisms underlying this dysregulation, and the potential role of microRNAs (miRNA) in these effects.

### 1.2. The Hypothalamus as a Central Homeostatic Regulator

The hypothalamus is a small region of the brain composed of distinct cell populations that play a crucial role in maintaining whole body homeostasis by regulating energy balance, reproduction, and circadian rhythms [14], among other processes. The hypothalamus, in part, links the central nervous system to the endocrine system, as neurons in the hypothalamus communicate with the pituitary gland by releasing hormones into the hypophyseal portal vein [15]. The secreted hormones will then induce or inhibit the secretion of hormones from the pituitary gland. The position of the hypothalamus allows it to sense nutrients, including palmitate, and peripheral signals that pass through the median eminence, a porous region of the blood brain barrier [15].

The hypothalamus is able to orchestrate multiple processes as a result of the heterogeneous populations of neurons that secrete neuropeptides with distinct functions. This inherent heterogeneity makes studying the direct effects of compounds on specific neuronal subpopulations difficult and identifying the underlying mechanisms nearly impossible. Primary cultures permit the study of direct effects of compounds on neurons, but these cultures have short life spans, limited numbers of surviving neurons, and are still heterogeneous in nature [16]. To resolve this, the Belsham lab generated a bank of clonal immortalized hypothalamic cell lines from murine, embryonic and adult hypothalamii. The immortalization process utilized primary cultures of hypothalamic neurons, the addition of ciliary neurotrophic factor (CNTF) to induce proliferation in adult neurons, and transformation with SV40 T-antigen [17]. The immortalized cells underwent selection with geneticin and were subsequently subcloned to generate genetically identical clonal populations that represent a single subpopulation of hypothalamic neurons. Immortalized heterogeneous neuronal populations of neuropeptide subtypes such as mHypoA-POMC/GFP, mHypoA-NPY/GFP, mHypoA-GnRH/GFP, and mHypoA-Kiss/GFP were also generated from the hypothalamii of transgenic mice containing enhanced green fluorescent protein (eGFP) downstream of the promoters of the genes of interest. The cells were sorted using fluorescent activated cell sorting (FACS), thereby selecting all immortalized neurons expressing GFP and as a result the neuropeptide-expressing neurons of interest [16]. These cell models have been used extensively to characterize neuronal function and the molecular events upon sensing cellular signals, such as palmitate [18].

### 1.3. Mechanisms and Biogenesis of microRNAs

microRNAs (miRNAs) are short (21–23nt) non-coding strands of RNA that complex with and act as guides for the argonaute (AGO) protein to regulate gene expression post-transcriptionally. They target gene transcripts based on complementarity to a 6-nucleotide seed sequence, which is often found at positions 2–7 from the 5′ end of the miRNA [19]. Upon binding to the targeted messenger RNA (mRNA), miRNAs can either inhibit the translation of the mRNA or lead to its degradation. This mechanism allows for the precise control of gene regulation by the cell and can be drastically altered in disease states [20].

The canonical pathway for miRNA synthesis begins with transcription by RNA polymerase II, which transcribes the primary miRNA (pri-miRNA) containing a stem loop structure with the miRNA sequence. After transcription, the pri-miRNA undergoes further processing within the nucleus by Drosha, a nuclear RNase III, which cleaves the pri-miRNA to release the stem-loop structure, known as the pre-miRNA [21]. The pre-miRNA is then exported to the cytoplasm by exportin 5, a ubiquitously expressed exportin that recognizes the double-stranded RNA portion of the stem-loop structure [22]. Once in the cytoplasm, the pre-miRNA is recognized by Dicer, a cytoplasmic RNase III, which cleaves the miRNA duplex from the stem-loop structure [23]. The miRNA duplex is then combined with AGO to form the RNA-induced silencing complex (RISC) and will concomitantly be used to target mRNAs for inhibition.

In addition to the actions that miRNAs have on the cells that produce them, they are also present in the circulation and can affect distant organs. miRNAs are transported through extracellular fluids within exosomes or bound to proteins. Exosomes are membrane-bound extracellular vesicles with a diameter below 100 nm. Interestingly, the quantity and content of exosomes are disrupted in disease states [24], suggesting miRNAs have potential for use as biomarkers [25]. Furthermore, exosomes enriched with miRNAs linked to the development of glucose intolerance and insulin resistance are detected in the blood of obese individuals [26,27]. The injection of exosomes collected from the adipose tissue macrophages of obese mice into lean mice caused glucose intolerance and insulin resistance, whereas the reverse improved insulin sensitivity in obese mice [28]. Taken together, these results suggest that secreted miRNAs present in exosomes likely mediate disease processes.

miRNAs have been predicted to play a pivotal role in gene regulation, as studies have estimated that as many as 60% of protein coding genes in the human genome may be targeted by miRNAs [29]. Furthermore, miRNAs are important for survival, as the global knockout of Dicer or DGCR8, a cofactor for Drosha function, leads to embryonic mortality [30,31]. The conditional knockout of Dicer in the mouse brain or in hypothalamic POMC neurons leads to hyperphagic obesity [32,33], demonstrating the importance of miRNAs in energy homeostasis. The dysregulation of miRNA expression in disease states indicates distinct miRNA expression profiles for individuals with obesity, type 2 diabetes mellitus, and cancers [34,35,36]. Of interest to this review, multiple miRNA array studies have shown that HFD exposure in mice can induce distinct miRNA expression profiles in the hypothalamus [37,38]. With the emerging potential of miRNAs to be used as biomarkers and therapeutics, studies exploring the physiological effects of miRNAs in the hypothalamus may provide a new class of tools to diagnose and target hypothalamus-associated metabolic disease. Hence, this review will include a perspective on miRNAs involved in hypothalamic function, and how palmitate exposure may alter these mechanistic pathways.

## 2. Energy Homeostasis

Energy homeostasis is primarily controlled in the arcuate nucleus (ARC) of the hypothalamus by two opposing neuronal populations, the orexigenic, appetite inducing, neuropeptide Y (NPY)/agouti related peptide (AgRP) expressing neurons and the anorexigenic, appetite suppressing, proopiomelanocortin (POMC) expressing neurons. The ARC is situated directly above the median eminence allowing it to sense peripheral signals and nutrients in the circulation. These neuronal populations synthesize and secrete their respective feeding neuropeptides in response to nutritional status communicated via peripheral hormones, nutrients and signaling molecules. The neuropeptides primarily act on second order neurons in the paraventricular nucleus (PVN) and the opposing neuronal population in the ARC to control energy homeostasis. NPY and AgRP induce feeding and inhibit the activity of POMC neurons [39], whereas POMC neurons, secreting α-melanocyte stimulating hormone (α-MSH) have the opposite effect, suppressing food intake and inhibiting NPY/AgRP neurons by activating melanocortin receptors, MC3R and MC4R [39]. This system is drastically altered in obesity as the neurons become resistant to peripheral satiety signals, such as insulin and leptin [40], leading to excessive food intake, weight gain, and metabolic disease [41].

### 2.1. Hypothalamic Insulin Signaling Is Impaired by Palmitate

#### 2.1.1. Insulin Signaling in the Hypothalamus

Although initially debated, the critical role of hypothalamic insulin action in regulating whole body energy homeostasis is now uncontested [42,43]. Pancreatic beta cells are the primary source of insulin; they secrete insulin in response to elevated blood glucose levels following a meal, allowing peripheral tissues to utilize or store the glucose [44]. Insulin travels through the circulation and is sensed by the hypothalamus at the median eminence [43]. Insulin is an anorexigenic hormone, and its hypothalamic actions lead to reduced food intake, and ultimately body weight, in rodents and primates [45,46]. Besides these anorexigenic effects, central insulin signaling in AgRP neurons is essential for suppression of hepatic gluconeogenesis [47], and in POMC neurons, it is necessary for the insulin-induced suppression of lipolysis in adipose tissue [48]. These actions are mediated by the ability of insulin to suppress orexigenic NPY/AgRP, while promoting anorexigenic POMC/α-MSH [49,50,51], which act on second-order neurons located in the PVN and other hypothalamic nuclei. Ultimately the actions of insulin in the hypothalamus and peripheral tissues achieve the same goal, which is to ensure glucose homeostasis after a meal [44].

**Figure 1:**
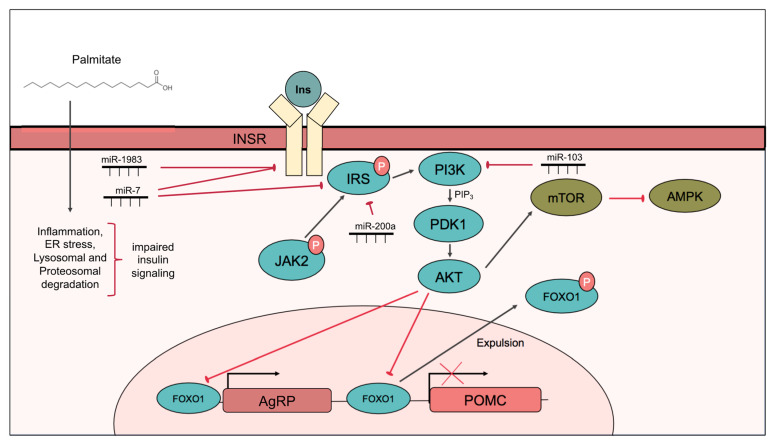
Hypothalamic insulin signaling. Insulin binds to insulin receptor on the cell membrane, leading to the phosphorylation of IRS, which in turn activates PI3K, to generate PIP_3_ that activates Akt via PDK1. Akt phosphorylates FOXO1 leading to its expulsion from the nucleus, leading to decreased *Npy* and *Agrp* and increased *Pomc* expression. Akt also activates mTOR signaling. Palmitate impairs insulin signaling by inducing, inflammation, ER stress, and lysosomal and proteasomal degradation of INSR or IRS. Components of the insulin signal transduction pathway are targeted by miRNAs include. INSR is targeted by miR-1983 and miR-7, IRS is targeted by miR-7 and miR-200a, and PI3K is targeted by miR-103.

Mechanistically, insulin acts via the insulin receptor (InsR), which dimerizes upon insulin binding and undergoes autophosphorylation of tyrosine residues, followed by recruitment and activation of insulin receptor substrate (IRS) proteins. Insulin actions occur primarily through two downstream signaling pathways. The first pathway mediates the effect of insulin on metabolism and begins with the activation of phosphoinositide 3-kinase (PI3K) by IRS. PI3K assists in the synthesis of PIP_3_, and PIP_3_ activates Akt via PDK1. Akt then phosphorylates forkhead box protein O1 (FOXO1) causing its expulsion from the nucleus, resulting in the anorexigenic actions of insulin signaling, which is to suppress *Npy* and *Agrp* and promote *Pomc* transcription. Akt also activates the mTOR signaling pathway, leading to the inhibition of AMP-activated protein kinase (AMPK), a protein kinase that is activated in low energy states to replenish ATP levels (Figure 1). The second pathway mediates the effect of insulin on cell proliferation and differentiation and involves mitogen-activated protein kinase kinase(MEK)/extracellular signal-regulated kinase (ERK)1/2 activation [44]. A commonality between insulin and leptin signaling is PI3K, which integrates anorexigenic signals from both pathways [52] and is discussed further later in this review.

#### 2.1.2. Hypothalamic Insulin Resistance

A hallmark of pre-diabetes and type II diabetes is an inability to respond appropriately to insulin. This phenomenon, known as insulin resistance, results from chronically elevated insulin levels, termed hyperinsulinemia, and desensitization of the insulin signaling pathway, ultimately resulting in improper energy balance. High calorie diets induce constant insulin secretion and activation of InsR, leading to its downregulation as well as desensitization of downstream signaling molecules [43,44]. Excess insulin-induced insulin resistance has been demonstrated in several hypothalamic neuronal models, including NPY/AgRP- and POMC-expressing neurons [51,52,53]. Specifically, insulin treatment in the mHypoE-46 cell line, an NPY/AgRP-expressing model, downregulated *Npy* and *Agrp* expression in the immediate term, as expected [50]. However, high insulin exposure (100 nM) for as little as 8 h downregulated IRβ, IRS1 and diminished subsequent insulin-stimulated Akt phosphorylation [53]. mTOR-S6K1-mediated phosphorylation of IRS-1, and degradation of IRS-1 and InsR via the proteasomal or lysosomal pathway, respectively, were implicated in the mechanism of insulin-induced insulin resistance in these NPY/AgRP-expressing cells [53].

#### 2.1.3. Induction of Central Insulin Resistance by Palmitate

Saturated fats can also directly induce insulin resistance in peripheral and hypothalamic cells in the absence of high insulin levels. Palmitate induces inflammatory responses, facilitated by nuclear factor kappa B (NFκB) activation, which is the primary mediator of HFD-induced insulin resistance in peripheral tissues, including adipocytes, muscle and liver [54]. Given evidence that hypothalamic neuroinflammation occurs within one day of HFD exposure, prior to inflammation in peripheral tissues, [55] and central insulin signaling is required for peripheral insulin action [42,47,48], fat-induced hypothalamic insulin resistance merits attention. In fact, intracerebroventricular (ICV) administration of palmitate impairs hypothalamic insulin signaling and leads to disruptions in hepatic glucose production and peripheral glucose metabolism in rodents [56,57]. Knockdown of protein kinase C theta in the ARC or toll-like receptor 4 (TLR4)-adaptor molecule, MyD88, in the CNS prevented HFD- or ICV palmitate-induced weight gain and insulin resistance [56,57], highlighting some of the mechanisms of palmitate-induced central insulin resistance. In the NPY/AgRP-expressing mHypoE-44 cell line, palmitate pre-treatment diminished the response to an insulin challenge, as demonstrated by reduced pAkt activation [58]. This did not occur in POMC-expressing mHypoA-POMC/GFP-1 cells [59], suggesting a protective mechanism in the POMC-expressing cells. The proinflammatory cytokine tumour necrosis factor alpha (TNFα) also induces insulin resistance in NPY/AgRP neurons [60]. Thus, along with the involvement of MyD88, a molecule that is essential for the generation of pro-inflammatory cytokines [57], these results strongly implicate neuroinflammation as a mediator of palmitate-induced insulin resistance. In contrast to palmitate, Amine et al. recently described that the polyunsaturated fat docosahexaenoic acid (DHA) did not induce pro-inflammatory cytokine production nor lead to hypothalamic insulin resistance in a human neuroblastoma cell line [61]. Our studies in vitro also suggest that DHA is protective against TNFα-induced neuroinflammation through GPR120 [62], implicating DHA as a protective fatty acid against the effects of palmitate.

Alternative mechanisms by which palmitate can induce insulin resistance include AMPK inhibition and ER stress. Treatment with an AMPK activator, aminoimidazole carboxamide ribonucleotide (AICAR), prevented palmitate-induced phosphorylation of cJun N-terminal kinase (JNK) and restored insulin signaling in the mHypoE-44 cells [58]. Indeed, activation of AMPK prevented hyperglycemia in insulin-resistant, leptin-deficient mice [63], and improved glucose tolerance in insulin-resistant Zucker rats [64]. This protective phenomenon is thought to result from the ability of AMPK to increase fatty acid oxidation, thereby decreasing fatty acid levels [65], or to inhibit mTOR signaling, as overactive mTOR is related to insulin resistance [66]. ER stress, resulting from the accumulation of misfolded proteins in the ER lumen, is also induced with palmitate and may play a role in palmitate-mediated hypothalamic insulin resistance [43,58]. To summarize, neuroinflammation, AMPK inhibition/mTOR activation and ER stress are involved in the mediation of hypothalamic insulin resistance by palmitate [43]. These processes involve signaling proteins that converge on pathways activated by insulin, resulting in modified signaling. As an example, palmitate upregulates SOCS3, as a result of nuclear factor κ B (NFκB) activation, subsequently leading to IRS1 degradation and prevention of InsR auto-phosphorylation. Inhibition of the NFκB pathway with PS1145 in turn reduced food intake and diminished hypothalamic insulin resistance in mice fed a HFD [67], identifying a targetable pathway to restore hypothalamic insulin signaling.

#### 2.1.4. Role of miRNAs in Hypothalamic Insulin Signaling and Resistance

The role of miRNAs in mediating insulin signaling and resistance and their potential ability to therapeutically prevent or reverse insulin resistance is an area of increasing interest. Of note, there is evidence hypothalamic miRNAs play an important role in insulin action and whole-body energy homeostasis. Firstly, hypothalamic knockout of Dicer leads to overactivation of the PI3K/Akt/mTOR pathway, akin to the effects of high insulin levels, suggesting miRNAs may serve to prevent overactivation of insulin signaling [68]. Specifically, miR-103 administration to mice lacking hypothalamic Dicer prevented overeating and obesity [68]. miR-103 targets two components of the insulin signaling pathway: Pik3cg, a catalytic subunit of PI3K, and IRS1 [68,69] (Figure 1). Overexpression of Lin28a in the hypothalamus improved glucose tolerance and insulin sensitivity in HFD-fed mice, suggesting the importance of hypothalamic miRNAs in whole body insulin sensitivity [70]. Components of insulin and leptin signaling often crosstalk and therefore miRNAs can affect both pathways. An example of this is miR-200a, a miRNA upregulated in the hypothalamus of ob/ob mice and targets both IRS2 (Figure 1) and LepR in the hypothalamus [71]. ICV administration of miR-200a antagomir in ob/ob mice increased the expression of insulin receptor in NPY and POMC neurons [71]. Because miRNAs can target hundreds of genes, there resides a potential symphony of intracellular events that target multiple pathways, ultimately leading to metabolic disease.

Two miRNAs, miR-1983 and miR-7, have emerged as miRNAs associated with neuronal insulin resistance that directly target the InsR (Figure 1). Treatment of mHypoE-46 neurons with high levels of insulin for 24 h identified miR-1983 as a candidate that is induced with insulin resistance in hypothalamic neurons. Exposure to miR-1983 mimics downregulated InsR β-subunit protein levels and target analysis identified a binding site for miR-1983 in the 3′UTR of IRβ [72]. miR-1983 levels were also increased in the hypothalamus of MKR mice [72], a non-obese model with impaired insulin signaling and hyperinsulinemia [73], and was positively correlated to insulin and homeostatic model for insulin resistance (HOMA-IR) scores in human serum samples, representing their potential for use as a biomarker. miR-7 is a miRNA that is highly abundant in the hypothalamus, along with let-7c and miR-9 [74]. miR-7 was elevated in mice fed a HFD, and insulin induced the miR-7-expressing intron and its parent gene heterogeneous nuclear ribonucleoprotein K. Interestingly, miR-7 targets multiple components of the insulin signaling pathway, including the InsR, IRS2, and insulin-degrading enzyme (IDE) [75], suggesting potent downregulation of insulin signaling in the presence of this miRNA. However, knockdown of miR-7 in POMC neurons of female mice exacerbated diet-induced obesity [76], suggesting that the neuronal subtype and sex differences play an important role in the function of miR-7. Antagonizing both miR-1983 and miR-7 in specific neuronal subtypes may have therapeutic potential as preventing the downregulation of the InsR is one potential way to combat hypothalamic insulin resistance. Future studies investigating the combined effects of several of these insulin-related miRNAs may provide avenues for combating multiple aspects of central insulin resistance.

### 2.2. Leptin Signaling Is Impaired by Palmitate

Leptin is an anorexigenic circulating hormone involved in the hypothalamic control of appetite. It is predominately synthesized in adipocytes and secreted into the circulation. The circulating concentration of leptin increases acutely after a meal to suppress appetite and the levels are proportional to the amount of fat mass [77,78]. Mutation of the leptin receptor (LepR), whereby a longer defective variant is expressed, causes hyperphagia, leading to obesity [79]. The restoration of leptin signaling in the central nervous system alone is able to restore the appetite suppressing effects of leptin and reverse the obese phenotype in LepR-deficient mice [80]. Obese individuals typically have chronically elevated levels of circulating leptin as a result of greater fat mass [78]. Despite this, the anorexigenic effects of leptin is lost in obese individuals, a phenomenon known as leptin resistance [81]. The primary cause of leptin resistance has not been identified but has been hypothesized to be caused by either impaired leptin transport across the blood brain barrier or the continuous activation of a negative feedback loop in the leptin signaling pathway [82,83].

#### 2.2.1. The Mechanisms of Hypothalamic Leptin Signaling

**Figure 2:**
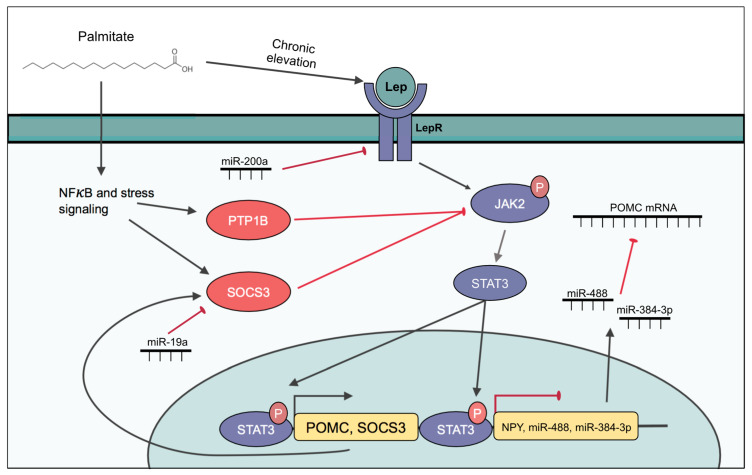
Hypothalamic leptin signaling. Leptin binds to LepR on the cell membrane, inducing the phosphorylation of JAK2, which in turn phosphorylates STAT3, and causes the translocation of STAT3 to the nucleus. In the nucleus, p-STAT3 induces the expression of *Pomc* and *Socs3* and represses the expression of *Npy*, miR-488, and miR-384-3p. SOCS3 negatively feeds back to inhibit JAK2 phosphorylation. miR-488 and miR-384-3p target the 3′-UTR of *Pomc* mRNA and inhibit its expression. miR-200a targets the 3′-UTR of *LepR*, inhibiting its expression and reducing leptin sensitivity. *Socs3* is targeted by miR-19a and can enhance JAK2-STAT3 signaling. Palmitate directly induces leptin resistance by inducing inflammation and ER stress, resulting in the activation of PTP1B and SOCS3. Excess palmitate can also induce leptin resistance by chronically increasing circulating leptin, leading to overactivation of the signaling pathway.

Leptin achieves its appetite suppressing effects primarily via LepR signaling in the ventromedial hypothalamus and the ARC, which are involved in the control of feeding [84]. In anorexigenic POMC expressing neurons, leptin induces the transcription of *Pomc* mRNA and the secretion of α-MSH [85,86]. In contrast, leptin suppresses the transcription and secretion of NPY and AgRP in orexigenic neurons [87,88]. Leptin signaling begins with the phosphorylation of janus kinase 2 (JAK2), which in turn phosphorylates signal transducer and activator of transcription 3 (STAT3), leading to its translocation to the nucleus, where it activates *Pomc* transcription and inhibits *Npy* and *Agrp* transcription [89,90,91,92]. Phosphorylated STAT3 also promotes the transcription of suppressor of cytokine signaling 3 (SOCS3), which in turn inhibits JAK2 activity [93], acting as a negative feedback loop to prevent chronic activation (Figure 2). Thus, a potential mechanism for the development of leptin resistance is the continuous transcription and activation of SOCS3 [93]. Leptin signaling can also crosstalk with the insulin signaling pathway, as JAK2 can lead to the phosphorylation of FOXO1 via IRS1/PI3K/Akt signaling [94]. The induction of insulin resistance in rat- and mouse-derived immortalized neurons also attenuates leptin signaling and its effects on gene expression [52]. Thus, there is a complex relationship between signaling components in neurons that require further investigation and disentangling of their roles in hypothalamic function.

#### 2.2.2. The Induction of Central Leptin Resistance by Palmitate

Leptin resistance is caused primarily by chronically high circulating levels of leptin. This has been demonstrated in the mHypoA-NPY/GFP cell line, an adult murine-derived *Npy*-expressing neuronal model, as an 8 h leptin exposure attenuates subsequent leptin-induced suppression of NPY secretion [95]. Hypothalamic exposure to saturated fatty acids has also been shown to induce leptin resistance. For example, ICV administration of palmitate in C57BL/6J mice led to central leptin resistance, as the appetite suppressing effects of leptin were lost and the phosphorylation of JAK2 and STAT3 were attenuated [96]. This coincided with an increase in the proinflammatory genes TNFα, interleukin 6 (IL6), and Interleukin 1 beta (IL1-β) [96]. Indeed, neuroinflammation has been implicated in the development of leptin resistance, as the activation of NFκB signaling, the hallmark regulator of proinflammatory cytokines, leads to an induction of SOCS3 and protein tyrosine phosphatase 1B (PTP1B), which are both negative regulators of leptin signaling [97,98]. Similar to neuroinflammation, ER stress induced by palmitate can lead to leptin resistance via PTP1B, and conversely, relieving ER stress with chemical chaperones, increased leptin sensitivity in diet-induced obese mice [99,100,101]. Overall, these findings demonstrate that exposure to palmitate can directly induce central leptin resistance via modifying proteins involved in leptin signaling.

#### 2.2.3. The Role of miRNAs in Leptin Signaling and Resistance

The role of miRNAs in leptin signaling is still a new area of investigation, but the importance of their involvement has been established. The conditional knockout of Dicer in POMC neurons, resulted in increased leptin sensitivity, evident from the greater suppression of food intake by leptin and a reduction in food intake overall [102]. Furthermore, miR-200a and miR-200b have been shown to directly target leptin mRNA in yellow catfish [103]. In mice, miR-200a targets the 3′-UTR of LepR and has been shown to be regulated by leptin itself, as mice with deficient leptin signaling have increased hypothalamic miR-200a expression and central leptin administration decreases it [71]. Therefore, an induction of miR-200a by exogenous compounds, including dietary fats, may lead to reduced leptin sensitivity and blocking mir-200a may serve as a tool to relieve leptin resistance. miRNAs can also target components involved in the leptin signaling pathway, such as SOCS3, which is targeted by miR-19a [104]. Administration of miR-19a can therefore enhance leptin sensitivity by relieving inhibition on JAK-STAT signal transduction and warrants further investigation. A group of conserved miRNAs have been shown to mediate the effects of leptin signaling in POMC neurons. The miRNAs of interest, miR-383, miR-384-3p, and miR-488, target the 3′ UTR of the *Pomc* mRNA in the mHypoA-POMC/GFP-1 cell line [105] (Figure 2). The expressions of these miRNAs in the hypothalamus were dependent on leptin, as they were downregulated in response to leptin administration and were increased in leptin-deficient ob/ob mice [105]. The potential role of miRNAs in mediating leptin response or resistance in NPY/AgRP-expressing neurons remains to be explored.

### 2.3. Feeding Neuropeptides

#### 2.3.1. Palmitate-Induced Changes in Neuropeptide Expression

In addition to modifying the response of hypothalamic neurons to peripheral signals, palmitate can also directly affect the expression of feeding neuropeptides. In the mHypoA-POMC/GFP-2 cell line, an adult murine derived *Pomc*-expressing neuronal model, exposure to palmitate increased *Pomc* mRNA expression and induced a myriad of neuroinflammatory and ER stress markers [59]. This induction of *Pomc* mRNA by palmitate was independent of neuroinflammation as neither inhibition of TLR4 nor NF𝜅B signaling were able to block the effects of palmitate, but it was dependent on palmitate metabolism to palmitoyl-coA and activation of MAP kinases, JNK and ERK [59]. Though the effects of palmitate on *Pomc* and inflammatory marker gene expression were not dependent on each other, the monounsaturated fatty acid oleate, which has been shown to block the effects of palmitate in multiple different tissues, blocked changes in the mRNA expression of *Pomc*, inflammatory and ER stress markers [59]. In the mHypoE-44 and mHypoE-46 cell lines, which are embryonic-derived *Npy*-expressing neuronal models, exposure to palmitate increased *Npy* mRNA expression, which was attenuated by PS1145, an inhibitor of IKK [106,107,108], illustrating a role for neuroinflammation in the alteration of *Npy* expression. The development of chronic, low-grade neuroinflammation in vivo has been suggested to play a significant role in altering feeding neuropeptide expression [106,109]. Studies in the mHypoE-46 cell line demonstrate that exogenous administration of TNFα and visfatin, proinflammatory cytokines induced by palmitate, induce *Npy* expression [106,110]. However, the exogenous administration of other palmitate-induced pro-inflammatory cytokines, Macrophage migration inhibitory factor (MIF) and IL-17F, were unable to affect *Npy* expression in the mHypoE-46 cell line [110]. Whereas PS1145 was only able to partially block the induction of *Npy* by palmitate [106], inhibition of acyl-coA synthetase completely blocks the increase in the mHypoE-46 cells [110], suggesting that the palmitate-mediated effects of *Npy* occur primarily through the metabolism of palmitate to ceramides and certain phospholipid species and is only partially dependent on neuroinflammation [110]. Furthermore, *Npy* dysregulation by palmitate in the mHypoE-44 cell line coincides with disruptions in the cyclic expression of circadian rhythm genes [108], including brain and muscle ARNT-like 1 (BMAL1), an essential mediator of palmitate-induced *Npy* upregulation [111]. This topic is discussed further in subsequent sections. *Agrp* mRNA expression is induced with palmitate in the mHypoE-41 cell line at 4 h and was blocked by siRNA-mediated knockdown of autophagy-related gene 5 (*Atg5*) [112]. In mHypoE-46 cells, palmitate induced *Agrp* mRNA expression at 16 h, which was blocked by metformin and salicylate treatment [107]. Furthermore, *Agrp* mRNA expression was unaffected by the proinflammatory cytokine TNFα, demonstrating the differential regulation of *Agrp* and *Npy* by pro-inflammatory mediators [107]. Taken together, palmitate is able to directly disrupt the expression of feeding neuropeptides in hypothalamic neurons via the induction of MAP kinases or inflammatory signaling, increasing palmitate metabolites, altering circadian transcription factors, and modifying autophagy.

#### 2.3.2. The Control of Feeding Neuropeptides by miRNAs

Research on miRNAs directly targeting feeding neuropeptides is severely limited. A total of three miRNAs have been identified to directly target the 3′ UTR of *Pomc* mRNA, miR-383, miR-384-3p, and miR-488 [105]. There are currently no published studies identifying miRNAs that directly target *Npy* or *Agrp*. miRNAs do not need to directly target a gene to affect its expression, as they can target components involved in the regulation of the gene, such as transcription factors and signal transduction molecules. Transcription factors involved in the regulation of *Npy* include cAMP response element binding protein (CREB), octamer transcription factor 1 (OCT1), and BMAL1 [113,114,115,116]. CREB, a transcription factor that positively regulates *Npy* expression, is targeted by several miRNAs, including miR-22-3p, miR-26a-5p, miR-27a-3p, miR-221-3p, miR-4474-3p, and miR-4717-3p [117,118]. A miRNA-induced decrease in CREB would likely lead to a downstream decrease in *Npy* expression. miR-155 has two binding sites in the 3′UTR of *Bmal1*, a transcription factor crucial for the rhythmic expression of *Npy* [119]. Furthermore, miR-155 is induced by inflammatory signaling and is increased in the adipose of obese humans [119,120]. A miRNA-induced decrease in *Bmal1* expression could result in the loss of rhythmic *Npy* expression, similar to what is seen in BMAL1-KO mice. Our lab recently identified miR-708-5p as a miRNA involved in *Npy* regulation, as transfection of the mHypoA-59 cell line with miR-708-5p mimic increased *Npy* mRNA [121]. This increase in *Npy* may be the result of miR-708-5p-mediated downregulation of its target neuronatin (NNAT), a putative sarco/endoplasmic reticulum Ca^2+^ inhibitor [122]. This hypothesis is supported by the fact that knockout of NNAT resulted in increased *Npy* expression in the ARC and an increased propensity to develop obesity in mice [123]. Transcription factors involved in the regulation of *Agrp* include Kruppel-like factor 4 (KLF4), activating transcription factor 3 (ATF3), STAT3, and FOXO1 [90,124,125,126]. KLF4, a transcription factor crucial for development and a positive regulator of *Agrp*, is targeted by two miRNAs, miR-206 and miR-145 [127,128]. The expression of miR-206 is increased 6-fold in the brain after a 20-week HFD [129], suggesting the potential involvement of miR-206 and KLF4 in palmitate-mediated *Agrp* induction. ATF3 is a stress induced transcription factor that induces *Agrp* transcription [130] and is targeted by at least three miRNAs, miR-27a-3p, miR-488, and miR-222 [131,132,133]. miR-222 is found in serum exosomes primarily produced by the gonadal white adipose tissue and is elevated in the serum of obese patients [134], implying a far-reaching mechanism by which palmitate can act. As with all miRNAs that target a positive regulator of a gene of interest, the miRNA-induced downregulation would cascade to a downregulation of the gene of interest. Thus, although a variety of miRNAs that target components involved in feeding neuropeptide regulation have been identified, there is still much to be done with respect to miRNAs that directly target *Npy*, *Agrp*, and *Pomc*, as many have been predicted to do so with in silico analysis.

## 3. Reproduction

Infertility occurs in 10% of women worldwide and often accompanies obesity [135]. Specifically, overweight and obese women are three times more likely to experience infertility [136,137] and the odds ratio of infertility increases with increasing body mass index (BMI) in men [138]. Even in cases of successful pregnancies, overweight and obesity is a main risk factor for gestational diabetes, occurring in up to 20% of pregnant women in Canada, the complications not only affecting the mother, but reaching adult offspring [139]. As such, consumption of an HFD impacts reproductive and offspring health, with evidence of excess fats disrupting multiple aspects of the hypothalamic-pituitary-gonadal (HPG) axis [136,140], the master regulator of reproductive function. In this section, we describe the effects of palmitate on the hypothalamic cells that initiate the HPG axis.

### 3.1. Hypothalamic-Pituitary-Gonadal Axis

Gonadotropin releasing hormone (GnRH) neurons of the hypothalamus are the main regulators of reproductive function, and disruptions to these neurons leads to infertility and improper development [141]. These neurons are primarily located within the medial preoptic nucleus of the hypothalamus but receive inputs from surrounding neuronal populations. At the anterior pituitary, GnRH induces the release of luteinizing hormone (LH) and follicle stimulating hormone (FSH) into circulation. LH and FSH then travel to the gonads, triggering the production and secretion of sex steroid hormones, estrogen, testosterone, and progesterone. The expression and secretion of GnRH are tightly regulated by afferent neuropeptides, satiety signals, hormones, and stress.

**Figure 3:**
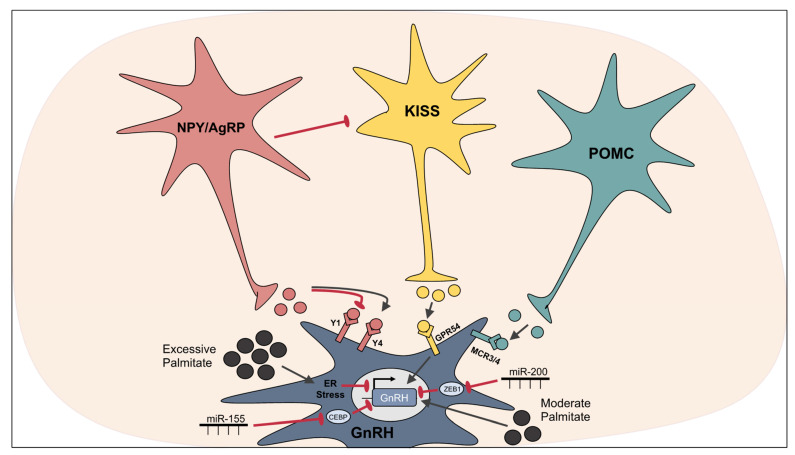
Regulation of GnRH neurons. NPY differentially affects GnRH neuron excitability depending on receptor variant availability; activation of Y1 is inhibitory and Y4 is excitatory. AgRP indirectly affects GnRH neurons by inhibiting afferent KISS1 neurons. KISS1 neurons secrete KISS1 which activates GPR54/KISS1r on GnRH neurons, leading to increased excitability and GnRH transcription. POMC neurons secrete α-MSH, which acts via MC3R and MC4R to increase GnRH neuron excitability. Moderate concentrations of palmitate induce *Gnrh* transcription, whereas high concentrations of palmitate induce ER stress and inhibit *Gnrh* transcription. miR-155 and miR-200 increase *Gnrh* mRNA by targeting *Gnrh* repressors CEBP and ZEB1, respectively.

Kisspeptin (KISS1) is a key reproductive peptide that regulates GnRH expression. The importance of KISS1 in reproduction cannot be understated as whole-body knockouts of KISS1 or its receptor, GPR54/KISS1r, lead to hypogonadotropic hypogonadism, a condition where little to no sex hormones are produced, resulting in infertility and the loss of puberty [142,143]. The role of KISS1 in reproduction can be traced to the hypothalamus as the conditional knockout of KISS1r in GnRH neurons or KISS1 in hypothalamic neurons is enough to induce an infertile phenotype [144,145]. KISS1 neurons are primarily located in the ARC and anteroventral periventricular nucleus (AVPV) of the hypothalamus allowing integration of nutrient signals into their actions [146]. ARC KISS1 induces the secretion of GnRH (Figure 3), leading to pulsatile LH and FSH secretion required for the onset of puberty and maintenance of reproductive function [147,148,149], while AVPV KISS1 evokes the pre-ovulatory GnRH and LH surge [141]. Phoenixin (PNX) is another recently discovered hypothalamic peptide that afferently controls GnRH expression. siRNA knockdown of *Pnx* in rats delayed the start of the estrous cycle by 2.3 days [150], highlighting its role in maintaining proper reproductive function. In hypothalamic neuronal models, PNX bound to GPR173 receptors and increased *Gnrh* mRNA, GnRH secretion, Gnrh receptor *(Gnrh-r)* mRNA and *Kiss1* mRNA [151]. GnRH-, KISS1- and PNX-expressing neurons are all subject to HFD- or palmitate-induced dysregulation as described below.

### 3.2. The Integration of Energy Homeostasis in the Control of Reproduction

Reproduction is a costly process that requires an appropriate nutritional status to succeed. As such, it is imperative that reproductive neurons integrate satiety signals from feeding neurons, including NPY/AgRP and POMC neurons. This requirement for proper energy homeostasis is emphasized by the fact that both malnourished and obese individuals often suffer from reproductive dysfunction [137,152].

Orexigenic NPY and AgRP signals are able to directly and indirectly affect GnRH neurons. NPY also suppresses KISS1 neuron activity in the ARC, thereby acting indirectly on GnRH through its afferents [153]. The overall directionality and magnitude of the effects of NPY on GnRH neurons is highly variable and dependent on the receptor subtypes present, NPY can activate or inhibit the reproductive axis. In contrast to the effects of NPY, the direct effects of AgRP on GnRH neuron excitability are minimal [154]. Instead, AgRP neurons may act on GnRH neurons indirectly via KISS1 neurons. Padilla et al. demonstrated that overactivation of AgRP neurons decreased fertility through a γ-aminobutyric acid (GABA)-dependent inhibition of KISS1 neurons [155]. The anorexigenic POMC neurons release α-MSH, which exerts an excitatory effect on GnRH neurons via MC3R and MC4R [154,156]. This excitatory effect of α-MSH on GnRH neurons may be a signal indicating sufficient nutrients are available for reproduction (Figure 3). Taken together, whether direct or indirect, communication between GnRH and feeding neurons is essential for proper reproductive function, and disruptions to feeding neurons as described previously may also impair reproductive function.

In addition to receiving input from feeding neurons, GnRH neurons also contain receptors for peripheral satiety signals, such as insulin and leptin, and as a result can become insulin- or leptin-resistant. However, the presence of insulin signaling in GnRH neurons seems to be dispensable as mice with conditional knockout of the InsR in GnRH neurons develop normally and are fertile [157]. This is similar for leptin signaling, as mice with conditional knockout of LepR in GnRH neurons are fertile but do have a slight delay in the onset of puberty [158]. Thus, these peripheral satiety signals may relay their signals primarily through feeding-related neurons that project onto GnRH neurons as discussed above. KISS1 neurons may also relay this information as circulating leptin levels are correlated with circulating KISS1 levels and leptin is able to increase *Kiss1* mRNA expression [159,160]. In addition to sensing peripheral hormones, GnRH neurons can also directly sense nutrients such as glucose [161] and fatty acids [9]. Overall, these studies demonstrate the ability of reproductive neurons to integrate energy status and draws attention to probable mechanisms of HFD- and palmitate-induced disruption of hypothalamic reproductive neurons.

### 3.3. Palmitate-Mediated Disruption of Hypothalamic Reproductive Control

As discussed, obesity in both men and women is associated with a greater incidence of infertility [136,138], however this association can be confounded by genetic and other lifestyle factors. Fertility complications in rodents consuming an HFD have delineated direct effects of an HFD on reproductive function. For example, female rats fed an HFD had increased incidences of irregular estrous cycles after just 4 months on the diet and showed accelerated follicle loss [162]. HFD-fed male mice had functional sperm but reduced sperm counts [163]. At the pituitary, the effects of HFD on LH secretion are highly variable, with some studies observing an increase in LH, whereas others reported a decrease or no change [164,165,166,167]. Nevertheless, the majority of studies identify some dysregulation of the HPG axis, resulting in detrimental effects on reproductive function.

GnRH neurons can directly sense fatty acids as illustrated by in vitro studies. In the mHypoA-GnRH/GFP cell line, exposure to 50 to 100 µM palmitate increased *Gnrh* mRNA, which was mechanistically linked to palmitoyl-coA synthesis and increased PI3K signaling [9]. In the GT1-7 cell line, treatment with 500 or 1000 µM palmitate decreased *Gnrh* mRNA and this effect was seemingly the result of ER stress, which is commonly observed among obese individuals [168]. These dose-dependent effects of palmitate demonstrate the fatty acid sensing capabilities of GnRH neurons: lower concentrations of palmitate likely signals that sufficient nutrients are available for reproduction, thereby increasing *Gnrh*, but higher concentrations cause cellular stress and dysfunction and dampen *Gnrh* as may be seen in obesity. Inflammatory cytokines have been reported to decrease *Gnrh* expression and may represent a mechanism of HFD-mediated impairment of the HPG axis [169]. For example, HFD-fed rabbits had decreased GnRH and KISS1r immunopositivity alongside increased microglial activation and hypothalamic inflammatory cytokine expression [170]. Furthermore, ovariectomized female C57BL/6J mice become obese, but are protected against changes in the HPG axis. This is remarkably accompanied by a lack of microglial activation and inflammatory cytokine production as well as an increase in anti-inflammatory IL-10 levels in the hypothalamii of these female mice despite the lack of ovarian estrogen. These studies strongly tie together hypothalamic inflammation and reduced *Gnrh* expression [171].

In addition to the direct effects of palmitate on GnRH neurons, palmitate can also alter the function of neurons that afferently regulate GnRH neurons, including feeding neuropeptide expressing- and PNX-expressing neurons. Treatment of the mHypoE-46 cell line with palmitate increased the mRNA expression of *Pnx*, which has been shown to positively regulate GnRH expression and function [172]. However, palmitate also reduced the expression of the PNX receptor, *Gpr173*, via a p38-mediated mechanism and prevented PNX-induced upregulation of pCREB [173], suggesting potential dampening of the HPG axis. Although *Kiss1* and *Kiss1r* expression is reduced in the hypothalamus of male mice fed a HFD for 19 weeks [174], the direct effects and mechanisms of palmitate action on KISS1 neurons remain to be studied. Overall, palmitate alters hypothalamic reproductive neurons via direct actions on *Gnrh* expression or through the regulation of its afferents. How these effects can be combatted to prevent fertility complications in obesity remains an important question.

### 3.4. miRNAs Involved in the Hypothalamic Control of Reproduction

miRNAs may become useful tools in the study of palmitate-induced dysregulation of reproduction as they are already being investigated as biomarkers of infertility and pregnancy outcomes [175,176]. The literature surrounding miRNAs specifically involved in the hypothalamic control of reproduction is still very limited, but their importance is evident as conditional knockout of *Dicer* in GnRH neurons or in the pituitary gland leads to infertility or severely reduced fertility, respectively [162,177]. A miRNA “switch” has been identified in GnRH neurons involving miR-155, which targets CCAAT-enhancer binding protein beta (CEBPß), a transcription factor that negatively regulates GnRH, and miR-200, which targets Zinc finger E-box binding homeobox 1 (Zeb1), another repressor of GnRH and its transcriptional activators [177]. Prior to the onset of puberty, these miRNAs are increased, leading to the downregulation of the targeted GnRH repressors, and ultimately lead to increased GnRH [177]. In addition, 16 other miRNAs have been identified by in silico analysis to directly target the 3′ UTR of GnRH but none have been experimentally validated [178]. Another miRNA that has been associated with the onset of puberty in the hypothalamus is miR-30b. miR-30b targets the 3′-UTR of makorin RING-finger protein-3 (MKRN3), a protein associated with the inhibition of the onset of puberty [179]. Central administration of target site blockers prevented the binding of miR-30b to the 3′-UTR of MKRN3 and delayed the onset of puberty in female mice [179]. As for KISS1, a recent abstract described a miRNA that directly targets the 3′ UTR of *Kiss1* and is increased in obese individuals with hypogonadism, but due to intellectual property reasons has not been disclosed [180]. Overall, investigation into miRNAs involved in the hypothalamic control of reproduction and how they are altered by palmitate holds potential for future intervention studies.

## 4. Circadian Rhythms

Circadian rhythms are 24-h patterns of biological activity that occur to synchronize homeostatic functions, including feeding, reproduction, stress, temperature control, blood pressure and hormone production, with the outside environment [181]. Disruptions to these rhythms in humans through shift work or through genetic polymorphisms of the genes responsible for maintaining these rhythms are linked to increased rates of obesity, metabolic syndrome and diabetes [182,183,184]. As such, disrupting circadian rhythms may promote obesity by altering metabolism of nutrients; however, dietary factors can themselves disrupt circadian rhythms [185]. This section will explore how circadian rhythms in the feeding centers of the hypothalamus are disrupted by palmitate and how these changes mediate the other downstream actions of palmitate, including neuroinflammation, neuropeptide dysregulation and potentially miRNA alterations.

### 4.1. Control of Circadian Rhythms by the SCN and other Hypothalamic Nuclei

**Figure 4:**
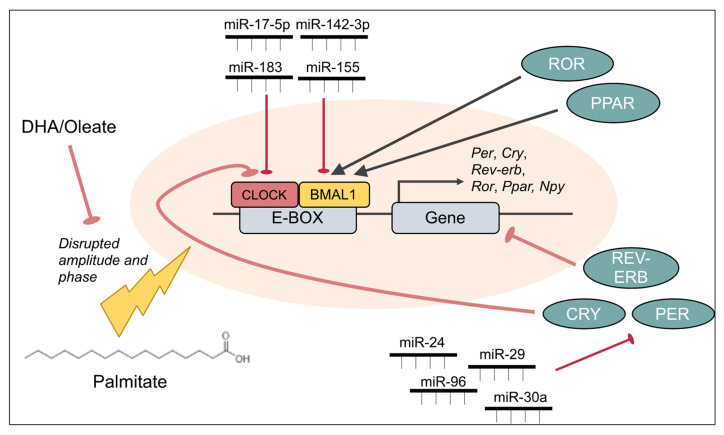
Circadian rhythm. The rhythmic expression of genes within a cell is controlled by a transcriptional-translational feedback loop primarily involving BMAL1, CLOCK, PER, and CRY proteins. The BMAL1 and CLOCK dimer binds to E-box sites in promoters to induce the expression of the downstream genes, which include *Per* and *Cry*. These negatively feedback onto the BMAL1:CLOCK dimer, thereby inhibiting their own transcription. Other circadian proteins involved in regulating the core elements of the loop include ROR, PPAR, and REV-ERB. Palmitate disrupts the rhythmicity of the loop, altering the amplitude or phase of rhythmic genes, including *Bmal1*, *Per2* and *Npy*. Core clock genes are also regulated by miRNAs. For example, *Bmal1* is targeted by miR-155 and miR-142-3p, *Clock* is targeted by miR-17-5p and miR-183, and *Pers* are targeted by miR-24, miR-29, miR-30a, and miR-96.

Circadian rhythms are generated internally by the suprachiasmatic nucleus (SCN) of the hypothalamus and are entrained or synchronized by zeitgebers or “time givers”, including light, food, body temperature and social cues [185]. The SCN is then able to control the rhythms of all other cells in the body via projections to other hypothalamic nuclei and brain regions [181,186]. At the molecular level, this occurs as a result of transcriptional-translational feedback loops involving clock genes, *Bmal1* and circadian locomotor output cycles protein kaput (*Clock*) that are transcribed and translated and act as transcription factors to positively regulate period (*Per1-3*), cryptochrome (*Cry1-2*), nuclear receptor subfamily 1,group D, member 1 (*Rev-erbs*) and retinoic acid-related orphan receptor (*Rors*) by binding to promoter elements called E-boxes. PER and CRY proteins then inhibit the action of BMAL1:CLOCK to prevent their own transcription, and REV-ERBs, RORs and peroxisome proliferator-activated receptors (PPARs) directly influence *Bmal1* transcription. This feedback loop creates a 24 h period where *Per* and *Cry* oscillate in an antiphasic manner to *Bmal1* expression [187]. These genes are subject to post-translational as well as post-transcriptional modifications (i.e., miRNA regulation) that contributes the length of the 24 h period [188,189,190] (Figure 4).

SCN rhythms are essential to maintain proper energy regulation and reproduction, as rodents with SCN lesions exhibit increased body weight, disrupted timing of food intake and activity [191], and a dysregulated LH surge and estrous cycle [192,193]. However, the SCN is not the sole player in circadian rhythms; the ARC and the PVN of the hypothalamus are core nuclei involved in food intake and energy expenditure [17], and synchronization of rhythms in these nuclei is critical to metabolic homeostasis. For example, SCN-ARC connections play a crucial role as microcuts removing this interconnectivity abolished rhythms in locomotor activity, corticosterone levels and body temperature in Wistar rats despite uninterrupted SCN rhythms [186]. Furthermore, deletion of BMAL1 in the PVN disrupts diurnal rhythms in metabolism and decreases neuronal response to refeeding, leading to obesity [194]. This dysregulation was attributed to perturbation of the rhythmic expression of the GABA-A receptor y2 subunit in the PVN neurons [194]. ARC neuropeptides NPY/AgRP and POMC show rhythmicity in vivo, with a peak in *Pomc* expression at 4 h after the dark phase [195], in *Agrp* expression in the transition between the light and dark phases, and in *Npy* expression once in the dark and once in the light phase [196]. These rhythms are also studied in vitro, where mechanisms of rhythmicity can be elucidated. Remarkably, the non-SCN, NPY/AgRP-expressing mHypoE-44 cell line demonstrated rhythmic expression of clock genes with an approximate period of 24 h [197]. Rhythmic binding of BMAL1 to the promoter region of *Npy* in these cells was associated with the rhythmic expression of *Npy* [197]. This ability of non-SCN hypothalamic cell lines to endogenously express rhythmicity has been recapitulated in murine adult-derived cell lines [111]. The importance of the molecular clock in NPY/AgRP-expressing neurons of the ARC is further demonstrated with the knockout of BMAL1 in AgRP-expressing neurons. These knockout mice demonstrated increased food intake with a trend towards increased body weight, a higher respiratory exchange ratio, and elevated hepatic glucose production [198]. Furthermore, coinciding with the transcriptional regulatory activity of BMAL1, the expressions of several genes were altered in AgRP-specific BMAL1-KO mice, including those involved in protein folding, secretory pathways, glucagon signaling, spliceosome activity and transcriptional response to leptin [198]. The observation that the major functions of AgRP-neurons were disrupted in these KO mice highlights the significance of BMAL1 and the circadian clock in NPY/AgRP neuron activity.

### 4.2. Palmitate-Induced Dysregulation of Circadian Rhythms in Hypothalamic Neurons

A major way in which the ARC and PVN neurons can desynchronize from the SCN is through exposure to exogenous factors, including high levels of fats and sugars [108,111,185,199,200,201]. Several in vivo studies have described hypothalamic neuropeptide expression and disruption of the rhythm of associated homeostatic processes, including body weight, food intake, and locomotion as a result of high fat diet exposure [199]. Consumption of a 45% HFD for 6 weeks dampened the diurnal pattern of food intake in C57BL6/J mice and although the expression profiles of hypothalamic clock genes were not altered, these mice displayed drastic changes in rhythmic *Npy*, *Agrp* and *Pomc* expression, indicative of HFD-induced disruptions in rhythmic feeding regulation at the level of the hypothalamus [199]. Moreover, although combined high-sugar, high-fat diets (termed a Western Diet (WD)) lead to obesity and disrupt circadian rhythms in food intake, the effects of a WD on hypothalamic neuropeptides or circadian proteins was not investigated until recently by our lab. Specifically, C57BL/6J mice exposed to a WD for 16 weeks showed altered diurnal feeding patterns of mice, with decreased food intake during the dark period and increased energy consumption during the light period [111]. These mice also displayed loss of rhythmic *Agrp* expression, a phase-shift in *Pomc* expression, and an overall reduction in *Npy* and *Agrp* expression in the hypothalamus [111]. In vitro experiments where neurons have been directly exposed to palmitate have indicated a causal relationship between palmitate, clock gene, and neuropeptide changes. For instance, palmitate-treated mHypoE-44 and mHypoE-37 neurons showed increased *Bmal1* expression and decreased *Per2* expression [108,200]. Likewise, palmitate-exposure in the mHypoE-44 neurons increased the amplitude of rhythmic *Npy* expression, which was mechanistically linked to AMPK activation by palmitate [108]. Thus, although causal links remained to be established, these studies strengthened the link between disruption of circadian clock genes, disruption of neuropeptides and disruption of whole-body homeostatic processes.

Remarkably, DHA, a ω-3 polyunsaturated fatty acid, prevented the palmitate-mediated upregulation in the amplitude of *Bmal1* expression in the mHypoE-37 cells [200]. DHA also led to a phase advance in *Bmal1* expression, however, DHA pre-treatment for palmitate-treated cells allowed normalization of the phase changed caused by both fatty acids. These results suggest potential protective actions of DHA against the saturated fatty acid palmitate in neurons and is corroborated by the recent finding of DHA-induced protection against fatty liver disease in HFD-fed mice through mechanisms involving circadian genes, RORα and REV-ERBα [202]. The monounsaturated fatty acid oleate has demonstrated protective effects against palmitate-induced changes in inflammatory markers and neuropeptides [9,59]. Interestingly, Tal et al. recently reported in 3T3-L1 adipocytes that palmitate increased the amplitude of clock gene expression, but decreased their overall expression after 24 h of treatment [203]. Oleate, in contrast, did not alter the amplitude of rhythmic expression nor lead to decreased expression. In fact, oleate increased the expression of *Clock* and *Cry1* [203]. Mechanistically, these differences were attributed to differential enzyme activation by oleate (AMPK) and palmitate (acetyl-coA carboxylase (ACC)). Palmitate also induced the activation of Akt and GS3Kβ, which both phosphorylate BMAL1 for exclusion from the nucleus of for ubiquitination [203,204,205]. Whether oleate has protective effects against palmitate-induced circadian dysregulation in the hypothalamus warrants investigation given the considerable ability of oleate to protect against neuropeptide and inflammatory dysregulation. These studies also highlight the potential mechanistic basis of palmitate-induced circadian dysregulation, where palmitate acts by disrupting proteins and enzymes that post-transcriptionally modify BMAL1 and the other circadian regulators to maintain proper rhythmicity.

To further establish the molecular clock as mediating the disruptive effects of an HFD or palmitate, peripheral as well as brain-specific BMA1-KO models have become useful tools. For instance, mice with BMAL1-KO in skeletal muscle resisted HFD-induced obesity due to increased oxidative capacity [206]. Microglial-specific BMAL1-KO also prevented diet-induced obesity due to increased microglial phagocytic capacity [207]. This ability led to increased retention of POMC neurons in the hypothalamus, which are typically selectively lost with exposure to an HFD [207,208]. Thus, brain-specific BMAL1 and circadian control are implicated as mediators of the effects of HFD-exposure. To establish links between BMAL1 and the direct effects of palmitate on hypothalamic neurons, our lab generated immortalized hypothalamic cell lines from male or female BMAL1-KO mice and their wildtype littermates, which served as appropriate controls [116]. Palmitate treatment in wild-type mixed population neurons (mHypoA-BMAL1-WT/F) or clonal NPY-expressing neurons (mHypoA-BMAL1-WT/8) increases *Npy* expression, while the respective BMAL1-KO counterparts failed to show increased *Npy* expression [111]. Mechanistically, palmitate treatment increases binding of BMAL1 to the promoter of *Npy*, indicating a causal link between palmitate-mediated *Npy* dysregulation and BMAL1 [111]. Furthermore, BMAL1 may play a role in the inflammatory actions of palmitate. In mHypoA-BMAL1-KO/F cells, palmitate increased basal *Il6* expression, but decreased basal *Nf𝜅b* expression. Moreover, the absence of BMAL1 altered the *Il6* and *Nf𝜅b* response to palmitate treatment [209]. To further highlight this inflammatory role, BMAL1 knockdown in microglial BV-2 cells led to an increased anti-inflammatory phenotype, and increased protection from palmitate-induced neuroinflammation [207]. As neuroinflammation has been established as a critical mediator of palmitate actions [55,59,107,210], the role of the circadian system in influencing this inflammation should not be ignored, particularly considering that in the mHypoE-37 neurons, circadian gene dysregulation with palmitate occurred prior to changes in inflammatory markers [200]. In contrast to knockdown of BMAL1, knockdown of REV-ERBα and REV-ERBβ specifically in tuberal hypothalamic nuclei (non-SCN) led to exacerbated weight gain and day-time food intake in male mice, but not female mice, fed an HFD; leptin insensitivity was implicated as the mediator [211]. These results coincide with the negative regulatory role of REV-ERBs on BMAL1 [185].

### 4.3. Role of miRNAs in Palmitate-Mediated Circadian Dysregulation

The role of miRNAs in the maintenance of normal circadian rhythms is demonstrated by whole-body knockdown of Dicer in mice. Dicer knockdown shortened the circadian period by 2 h as a result of faster translation of PER1 and PER2, due to the absence of three miRNAs that target PER: miR-24, miR-29 and miR-30a [212]. The role of miRNAs in palmitate-mediated circadian rhythm dysregulation can be thought of as two-fold. Firstly, miRNAs that target and control clock gene expression can be altered by palmitate, thereby altering the rhythmic expression of these genes and the transcriptional targets of clock genes. Secondly, miRNAs themselves can be rhythmically expressed, leading to specific fine-tuning and control of their targets. Palmitate-mediated alterations in these rhythmic miRNAs can influence a wide variety of gene products and this miRNA influence may underlie many of the palmitate-mediated changes in gene expression mentioned above. Palmitate-specific examples of both of these scenarios remain to be investigated in the hypothalamus; however, examples of both scenarios exist from other models [189,190]. For example, miR-142-3p in the SCN and miR-155 in bone marrow cells target BMAL1, both with two independent binding sites in the BMAL1 3′UTR [119,213,214]. Given that HFD-fed mice show reduced expression of miR-142-3p in muscle [215], it remains plausible that palmitate or an HFD may alter the expression of this miRNA in the hypothalamus. Furthermore, deletion of miR-155 protected HFD-fed female mice from developing obesity, accumulating white adipose tissue (WAT) and glucose tolerance, while it decreased WAT accumulation in males without any reduction in body weight [216]. Although this protection was associated with increased expression of brown adipose tissue promoting genes, the role of BMAL1 cannot be excluded [216]. Lastly, in a recent screen to identify rhythm-controlling miRNAs, 120 out of 989 miRNAs were identified to alter the period of *Bmal1-dluc* or *Per2-dluc* reporter containing cells. From this, miR-183/96/182 was followed up to be a conserved cluster and maintains the period and amplitude in human cells containing the *Per2-dluc* reporter, controls rhythmic locomotor activity, and targets *Per2* (miR-96) or *Clock* (miR-183) [217]. Interestingly, miR-96 was upregulated in the liver cells with palmitate exposure or with HFD exposure, targets IRS1 and insulin receptor and may contribute to fatty acid induced hepatic insulin resistance [218]. Whether its circadian function is also affected warrants investigation in these mice. Other miRNAs that have been reported to play a role in the murine SCN include miR-219, which is a target of CLOCK:BMAL1 [219], miR-132, which is responsive to light and fine tunes the clock by targeting chromatin remodeling [220], miR-17-5p, which itself displays a rhythm in the SCN via regulating CLOCK and is reciprocally regulated by CLOCK [221] (Figure 4). Any disruption to the rhythmicity of these miRNAs by palmitate has potential to alter the regulation of their targets. *Dicer* mRNA itself shows a diurnal rhythm of expression in the SCN, retina and bone marrow of mice, whereas this rhythm was phase-advanced in the SCN by aging and levels were decreased in the retina and bone marrow in diabetic animals [222]. Thus, the importance of rhythmic miRNAs in metabolic disorders warrants closer analysis and may prove therapeutically beneficial to fine-tune the expression of clock genes and their downstream targets.

## 5. Conclusions

Neurons in the hypothalamus control appetite, reproduction, and circadian rhythms and disruptions to these neurons have detrimental consequences for whole body health. Ultimately, these processes are interconnected and often undergo concurrent dysregulation. Thus, investigating the mechanisms of factors that dysregulate several of these functions will provide tools to combat their broad effects. Excessive exposure to saturated fatty acids such as palmitate, which are commonly found in HFDs, can disturb hypothalamic neurons resulting in resistance to insulin and leptin, dysregulation of feeding and reproductive neuropeptides, and disruptions of circadian rhythms that maintain energy homeostasis, and in turn reproduction. The effects of palmitate have been primarily linked to the induction of cellular stress, including neuroinflammation and ER stress, but avenues remain yet to be fully explored, including the actions of palmitate metabolites [110]. miRNAs play a key role in hypothalamic function and are dysregulated in disease states, and as a result, have become a novel and exciting area of study. Extracellular miRNAs can act as biomarkers to identify patients predisposed to certain metabolic conditions [25]. Furthermore, with the advent of technologies that enable packaging and therapeutic delivery of miRNAs to patients [223], they hold promise as an innovative tool to target key regulatory pathways in palmitate- and HFD-induced obesity and type 2 diabetes mellitus.

## Data Availability

Not applicable.

## Abbreviations

ACC	Acetyl-CoA carboxylase
AGO	Argonaute
AgRP	Agouti related peptide
AICAR	Aminoimidazole carboxamide ribonucleotide
AMPK	AMP-activated protein kinase
Arc	Arcuate nucleus
ATF3	Activating transcription factor 3
AVPV	Anteroventral periventricular nucleus
BMAL1	Brain and muscle ARNT-like 1
BMI	Body mass index
CEBPß	CCAAT-enhancer binding protein beta
CLOCK	Circadian locomotor output cycles protein kaput
CNTF	Ciliary neurotrophic factor
CREB	cAMP response element binding protein
CRY(1-2)	Cryptochrome (1-2)
CSF	Cerebrospinal fluid
DHA	Docosahexaenoic acid
eGFP	Enhanced green fluorescent protein
ER ERK	Endoplasmic reticulum Extracellular signal-regulated kinase
FACS	Fluorescent activated cell sorting
FOXO1	Forkhead box protein O1
FSH GABA	Follicle stimulating hormone γ-aminobutyric acid
GnRH	Gonadotropin releasing hormone
GNRH-R	Gonadotropin releasing hormone receptor
HFD	High fat diet
HOMA-IR	Homeostatic model for insulin resistance
HPG	Hypothalamus-pituitary-gonadal
ICV	Intracerebroventricular
IL1-β	Interleukin 1 beta
IL6	Interleukin 6
InsR	Insulin receptor
IRS	Insulin receptor substrate
IRβ	Insulin receptor beta
JAK2	Janus kinase 2
JNK	cJun N-terminal kinases
KISS1	Kisspeptin
KISS1r/GPR54	Kisspeptin receptor
KLF4	Kruppel-like factor 4
LepR	Leptin receptor
LH	Luteinizing hormone
MAPK	Mitogen activated protein kinase
MC3R	Melanocortin 3 receptor
MC4R	Melanocortin 4 receptor
MEK	Mitogen-activated protein kinase kinase
MIF	Macrophage migration inhibitory factor
miRNA	microRNA
MKRN3	Makorin RING-finger protein 3
mRNA	Messenger RNA
NF𝜅B	Nuclear factor kappa B
NNAT	Neuronatin
NPY	Neuropeptide Y
OCT1	Octamer transcription factor 1
pCREB	Phosphorylated cAMP response element binding protein
PER (1-3)	Period (1-3)
PI3K	phosphoinositide 3-kinase
PIP3	Phosphatidylinositol 3-phosphate
PKA	Protein Kinase A
PNX	Phoenexin
POMC	Proopiomelanocortin
PPAR	Peroxisome proliferator-activated receptor
pri-miRNA	Primary miRNA
PTP1B	Protein tyrosine phosphatase 1B
PVN	Paraventricular nucleus
Rev-erb	Nuclear receptor subfamily 1, group D, member 1
RISC	RNA induced silencing complex
ROR	Retinoic acid-related orphan receptor
SCN	Suprachiasmatic nucleus
SOCS3	Suppressor of cytokine signaling 3
STAT3	signal transducer and activator of transcription 3
TLR4	Toll like receptor 4
TNFα	Tumour necrosis factor alpha
UTR	Untranslated region
WAT	White adipose tissue
WD	Western diet
ZEB1	Zinc finger E-box binding homeobox 1
α-MSH	α-melanocyte stimulating hormone
